# Dynamics at the crystal-melt interface in a supercooled chalcogenide liquid near the glass transition

**DOI:** 10.1038/s41598-020-62783-5

**Published:** 2020-04-03

**Authors:** Jianheng Li, Rahul Jangid, Weidi Zhu, Chris Kohne, Andrei Fluerasu, Yugang Zhang, Sabyasachi Sen, Roopali Kukreja

**Affiliations:** 1 Department of Materials Science and Engineering, University of California Davis, 1 Shields Avenue, Davis, CA 95616 USA; 2 National Synchrotron Light Source II, Brookhaven National Laboratory, Upton, NY 11973 USA

**Keywords:** Glasses, Glasses

## Abstract

Direct quantitative measurements of nanoscale dynamical processes associated with structural relaxation and crystallization near the glass transition are a major experimental challenge. These type of processes have been primarily treated as macroscopic phenomena within the framework of phenomenological models and bulk experiments. Here, we report x-ray photon correlation spectroscopy measurements of dynamics at the crystal-melt interface during the radiation induced formation of Se nano-crystallites in pure Se and in binary AsSe_4_ glass-forming liquids near their glass transition temperature. We observe a heterogeneous dynamical behaviour where the intensity correlation functions g_2_(q, t) exhibits either a compressed or a stretched exponential decay, depending on the size of the Se nano-crystallites. The corresponding relaxation timescale for the AsSe_4_ liquid increases as the temperature is raised, which can be attributed to changes in the chemical composition of the melt at the crystal-melt interface with the growth of the Se nano-crystallites.

## Introduction

The structural relaxation during annealing near the glass transition region controls the properties and technological utility of glasses and/or glass-ceramics^1–4^. For example, glass products retain thermal stress caused by quenching, which decreases the strength and durability of the product and needs to be removed after forming via annealing^5^. This stress removal proceeds with structural relaxations through processes that are closely related to diffusion and viscous flow, with similar activation energy, at temperatures close to the glass transition. On the other hand, for glass-ceramics processing, the kinetics of nucleation and growth of crystals is controlled by the diffusion of atoms or larger structural units from the supercooled liquid to the crystal-liquid interface^6–8^. The standard kinetic models treat crystallization of a liquid as a macroscopic phenomenon and the applicability of these models is often hampered by the lack of a direct microscopic understanding of the atomic scale processes that accompany crystallization^9–11^. For example, these models use viscosity or diffusivity values for the bulk supercooled liquid, instead of the “local” microscopic transport property at the crystal-liquid interface, which is more relevant, albeit its experimental measurement would be rather difficult, if not impossible. Whether the transport or the relaxational timescale at the crystal-liquid interface can be treated the same as that of the bulk liquid is a key question that is still awaiting direct experimental confirmation.

X-ray photon correlation spectroscopy (XPCS) is a powerful experimental technique that offers the length scale and timescale resolution appropriate for the investigation of such atomic dynamics at the crystal-liquid interface. However, it has recently been shown that atomic dynamics can also be induced in non-metallic glassy systems by the incident x-ray beam, as it simultaneously pumps and probes the fluctuation dynamics^12^. Specifically, highly brilliant and coherent x-rays produced at third generation storage rings or free electron lasers can induce atomic movement, possibly through the process of radiolysis resulting in diffusion and long range rearrangement of atoms^13^.

In this article, we report the results of x-ray beam induced structural rearrangement leading to crystallization in a supercooled glass-forming liquid of pure Se and AsSe_4_ using XPCS. This technique allows us to capture coherent speckle patterns arising from fluctuations and inhomogeneities associated with the beam induced crystallization of supercooled glass-forming liquids close to their glass transition temperature *T*_*g*_. The intensity correlation function, which is related to the dynamical structure factor, exhibited either compressed or stretched exponential decay behaviour depending upon the size of the nano-crystallites. For crystallites larger than 70 nm, a compressed exponential behaviour was observed, while for smaller crystallites in the size range of 35–50 nm, a stretched exponential behaviour was observed, which is a hallmark of relaxation dynamics in supercooled glass-forming liquids as *T*_*g*_ is approached from above^14^. We observed an anomalous increase in the fluctuation timescale at the crystal-liquid interface as the temperature is increased for AsSe_4_, which likely resulted from a change in the local viscosity at the interface, due to local compositional changes. The results of the present study thus highlight the beam induced nanoscale nucleation and growth dynamics and how such dynamics are affected by crystallite size, in highly viscous supercooled glass-forming liquids.

## Results

**Figure 1:**
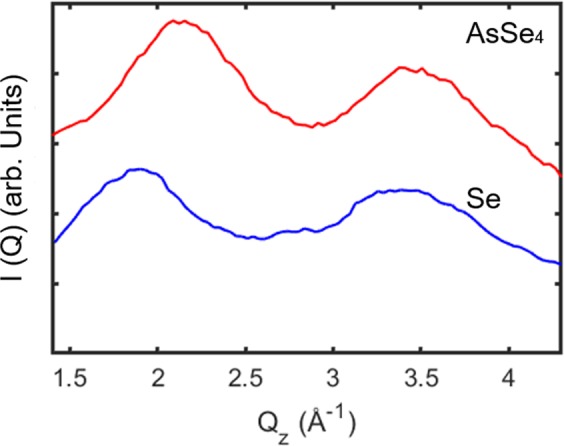
X-ray scattering intensity as a function of q for Se and AsSe_4_ at room temperature: The key features as well as the position of first maximum (principal peak) of static structure factor, Q_0_ = 1.8 Å^−1^ for Se and Q_0_ = 2.2 Å^−1^ for AsSe_4_ agree with the literature values.

**Figure 2:**
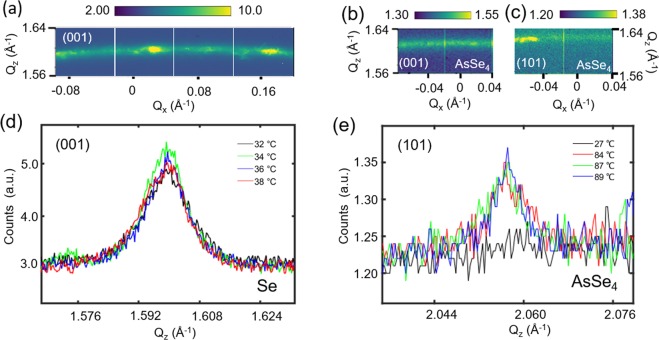
Polycrystalline diffraction rings observed from Se nano-crystallites in supercooled Se and AsSe_4_ liquid: Spotty polycrystalline rings observed for (**a**) Se sample at 38 °C, (**b**) and (**c**) AsSe_4_ samle at 89 °C, (**d**) compares the intensity of the (001) polycrystalline ring for a line cut along Qz for Se sample as a function temperature, and (**e**) compares the intensity of the (101) polycrystalline ring for a line cut along Qz for AsSe_4_ sample as a function of temperature.

X-ray scattering intensity as a function of q for pure Se and AsSe_4_ at room temperature is plotted in Fig. 1. For both samples, the key features as well the location of first maximum (principal peak) of the static structure factor, Q_0_ = 1.8 Å^−1^ for Se and Q_0_ = 2.2 Å^−1^ for AsSe_4_, agree with the literature values^15–17^. The coherent x-ray beam was tuned to the principal peak in symmetric Bragg geometry. X-ray beam induced crystallization of trigonal selenium was observed for both Se and AsSe_4_ samples as shown in Fig. 2. It may be noted here that these glasses do not display any detectable crystallization in the absence of radiation, for the same thermal treatments. Furthermore, the beam induced crystallization was also observed during XPCS measurements performed at a different x-ray energy, 7.35 keV, lower than Se edge. Similar evolution of time constant and correlation function was observed as shown in Supplementary Fig. S2, indicating that the crystallization is independent of x-ray energy.

Fig. 2(a)–(c) presents the polycrystalline rings observed near the principal peak for both Se and AsSe_4_ sample at Q = 1.61 Å^−1^ and 2.06 Å^−1^. Fig. 2(a) is measured at T = 38 °C, while Fig. 2(b),(c) are measured at T = 89 °C. Q_z_ represents the reciprocal vector parallel to the scattering plane and perpendicular to sample surface, while Q_x_ represents the reciprocal vector perpendicular to the scattering plane. Fig. 2(d),(e) depict the line cut through the polycrystalline ring along Q_z_ for Se at 32 °C, 34 °C, 36 °C and 38 °C and for AsSe_4_ at 27 °C, 84 °C, 87 °C and 89 °C, clearly demonstrating that the polycrystalline rings are absent near room temperature. This result suggests that the atomic motion associated with X-ray beam induced crystallization is a thermally activated process. The polycrystalline diffraction ring observed at the two different q-values correspond to the reflections from (001) and (101) lattice planes of Se^18^, indicating the formation of Se crystallites near Tg in both Se and AsSe_4_ samples. The calculated lattice parameters of a = 0.446 nm and c = 0.499 nm, agree well with the literature values reported for trigonal Se^6^. The spotty polycrystalline ring observed for Bragg reflections in both samples shows that the distribution of Se crystallites is not completely random and points towards the presence of preferred orientation. The Se crystallite sizes were estimated from the diffraction line widths using the Scherrer equation (see supplementary section 3 for details). A larger Se crystallite size in the range of 80–175 nm for Se sample and 70–105 nm for AsSe_4_ sample was obtained for the preferred orientation (higher intensity spots on the polycrystalline ring), irrespective of the temperature of measurements. These will be referred to as “large” crystallites in the subsequent discussion. On the other hand, for the random orientations (lower intensity regions), a Se crystallite size of ~37 nm was obtained at 32 °C, which increased to ~42 nm at 38 °C for Se sample and a Se crystallite size of ~35 nm was obtained at 84 °C, which increased to ~50 nm at 89 °C for AsSe_4_ sample. These will be referred to as “small” crystallites. We note that the speckles overlaying the diffraction peak in Fig. 2(d),(e), are a direct result of the disorder present in the sample and the degree of the coherence of the x-ray beam. The underlying dynamics in the sample near *T*_*g*_ manifest itself as the fluctuations of this speckle pattern.

In order to investigate these fluctuations, we measured the evolution of the speckle pattern as a function of time near *T*_*g*_. Fig. S3 in supplementary info show intensity vs. time “waterfall” plots or kymographs for the measured intensity. In order to quantitatively understand the evolution of small and large crystallites as a function of time, the intensity-intensity autocorrelation function *g*_2_*(q,t)* was calculated from the time-dependent speckle measurements using the relation:

1 $${g}_{2}(q,t)=\frac{ < I(q,t)I(q,t+\tau ) > }{ < I(q,t){ > }^{2}}$$

where *I(q,t)* and *I(q, t+τ)* are intensities of a given pixel separated in time by *τ* and <> denotes and ensemble average performed over all equivalent times tau and all equivalent pixels of the regions of interest considered here^19,20^. For the large Se crystallites, in both Se and AsSe_4_ samples, the *g*_2_*(q,t)* was calculated by averaging speckles in the high intensity region of the (001) or (101) polycrystalline ring only during the time window where the large crystallites are aligned to the Bragg condition. This procedure allowed us to remove any artifacts due to the sudden variation of intensity associated with the appearanceor disappearance of large crystallite in the XPCS scan. Detailed description of *g*_2_*(q,t)* calculation for large Se crystallites is provided in supplementary information section 5. The *g*_2_*(q,t)* for small crystallites was calculated by averaging speckles in lower intensity region of the (001) or (101) polycrystalline ring. The autocorrelation function calculated from the intensities is related to the intermediate scattering function (ISF), *|F(q,t)|*^2^, and can be fitted with a stretched exponential as given in Eq. (2).

2 $${g}_{2}(q,t)=1+A\,{e}^{-2{(t/\tau )}^{\beta }}=1+A|F(q,t){|}^{2}$$

**Figure 3:**
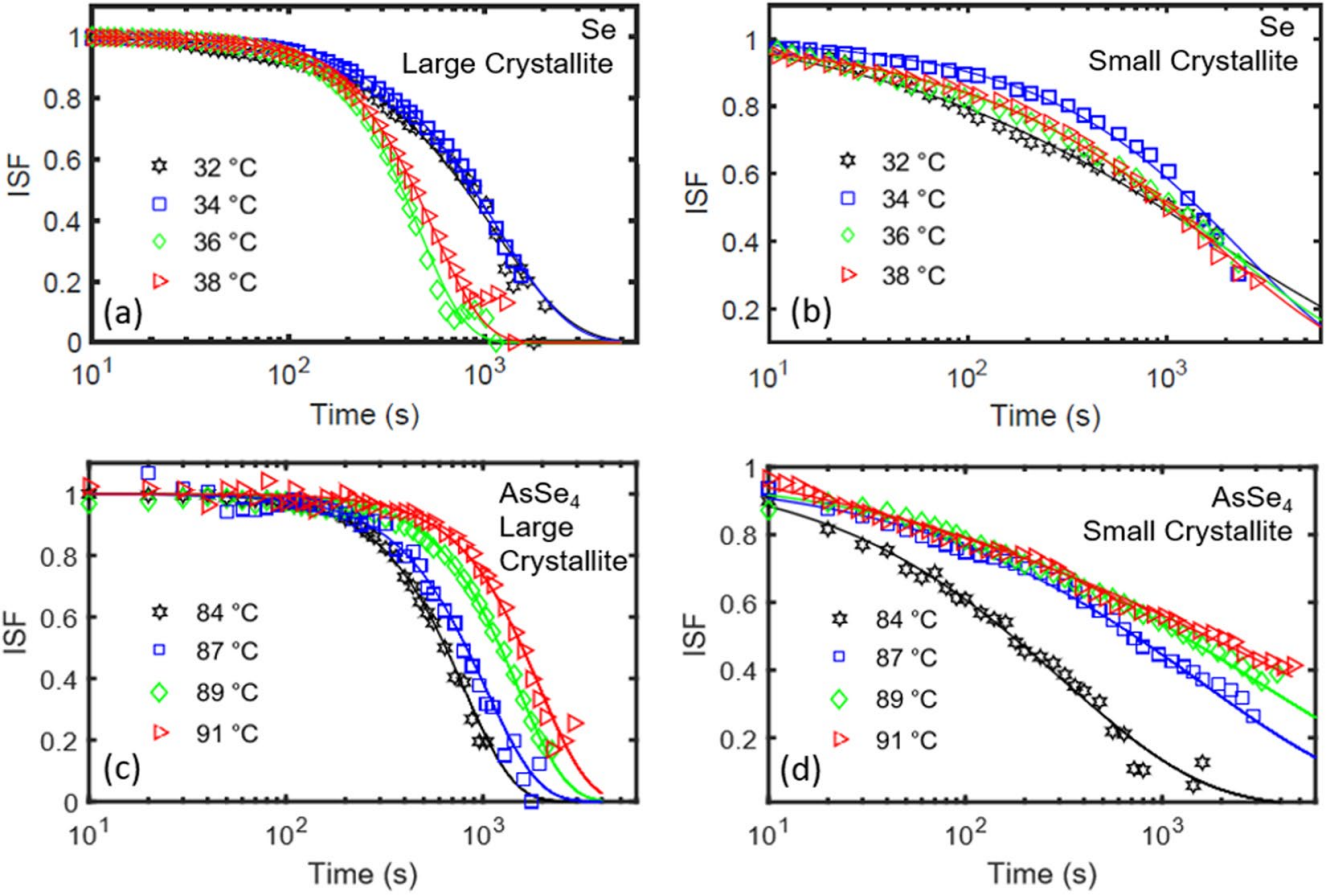
Evolution of ISF as a function of temperature: Experimental ISF data (symbols) and corresponding fits (lines) using Eq. 2 for (**a**,**b**) Se sample, (**c**,**d**) AsSe_4_ sample. ISF for large crystallites was calculated by averaging speckles in high intensity region of the polycrystalline rings, only during the time window where large crystallites are aligned to Bragg condition, and for small crystallites was calculated by averaging speckles in lower intensity region of the polycrystalline rings. For the large crystallites, compressed exponential is observed while for the small crystallites, stretched exponential is observed. The fits are based on Eqs. 1 and 2 as described in the text.

Here *A* is the speckle contrast, *τ* is the decay constant and *β* is the stretching exponent. Fig. 3 shows the temporal evolution of ISF for both Se and AsSe_4_ samples for temperatures ranging from 32 °C to 38 °C and 84 °C to 91 °C, respectively. Here we note that the ISF value of 1 represents fully correlated system while 0 represents complete de-correlation. While q-dependence due to variation in lengthscales has been observed in similar systems^21,22^, no significant q-dependence was observed here possibly due to the fact that the lengthscales near the two Bragg peaks represent similar d-spacing (0.1962 nm for (001) and 0.1543 nm for (101) Se Bragg peak). Thus q-dependence was omitted from the g_2_ and the ISF calculations. We also note that the ISF calculations for diffuse scattering near Bragg peak showed similar results and behaviour, albeit lower signal noise to ratio due to lower intensity. Furthermore, due to low speckle intensity resulting in small signal to noise ratio as well as observation of beam induced crystallization, structural relaxation dynamics of the bulk liquid near *T*_*g*_ could not be detected. No aging behaviour was observed as shown by two-time correlation plots and time-resolved measurements in Figs. S5–S7 of supplementary information

**Figure 4:**
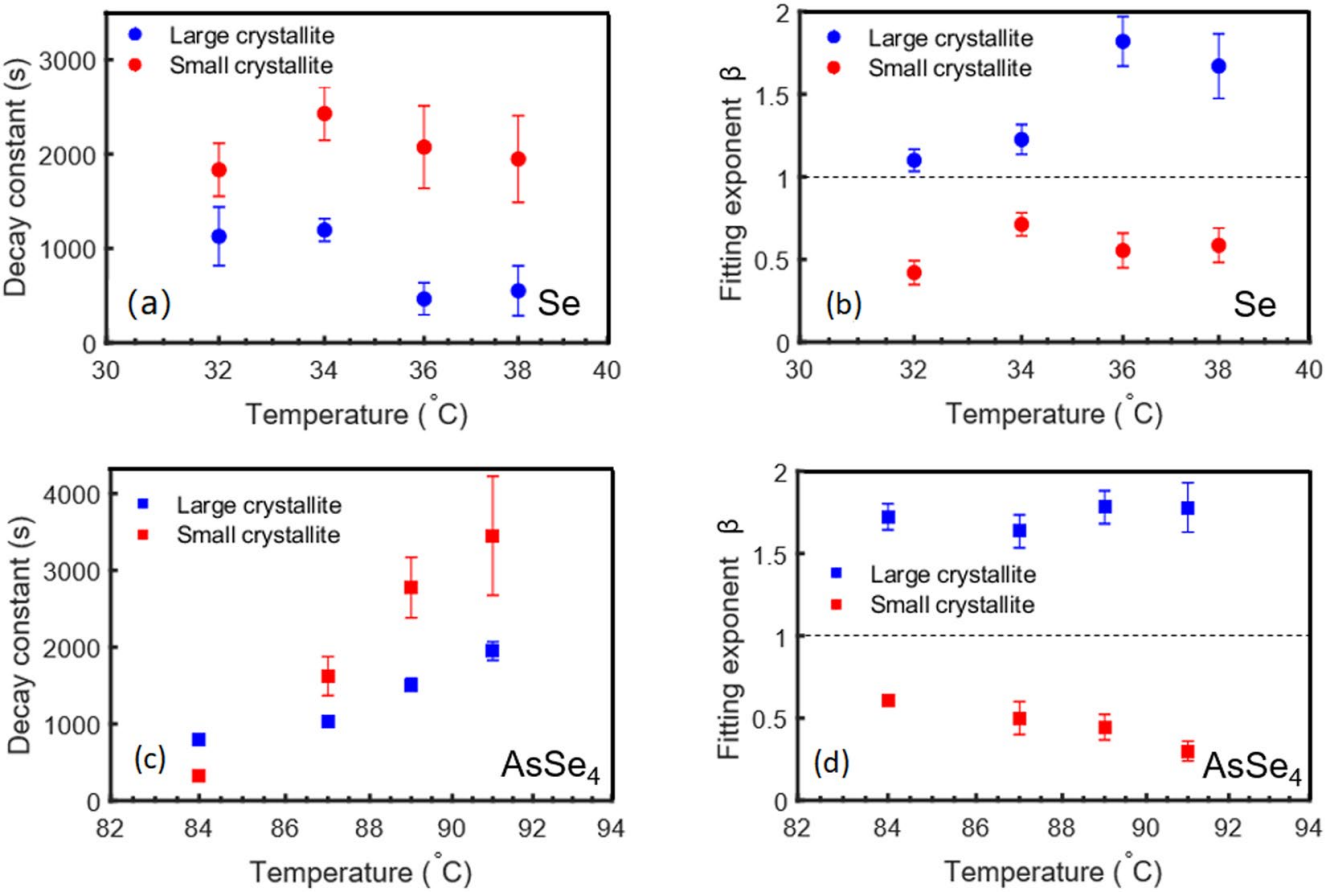
Characteristic timescales and exponent as a function of temperature: Characteristic fluctuation timescale (τ) and exponent (β) as a function of temperature, obtained from the fits of ISF for (**a,b**) Se sample, (**c,d**) AsSe_4_ sample.

Fig. 4 shows the values obtained for τ and β as a function of temperature for both samples. The error bars were calculated using the uncertainty in counting statistics for different Q_x_ regions. Significant temperature dependence of the fluctuation timescale was observed for both samples. For Se sample, a slight decrease in τ is observed for both large and small crystallites on increasing the temperature from 32 °C to 38 °C as shown in Fig. 4(a). This decrease in time constant is consistent with decrease in viscosity of a liquid resulting in higher fluctuations with increasing temperature. However, surprisingly, for AsSe_4_ sample, an opposite behaviour i.e. an increase in τ is observed for both large and small crystallites on increasing the temperature from 84 °C to 91 °C as shown in Fig. 4(c). The rise in τ is particularly dramatic for the small crystallites, where the timescale increases by nearly an order of magnitude over the measured temperature range. This increase in τ i.e. slowing down of fluctuations as a function of temperature is surprising and counterintuitive as the viscosity of a liquid is expected to decrease with increasing temperature^23^. The exponent β calculated for the large and small crystallites is shown in Fig. 4(b),(d). We find the exponent to be ~1.5 for the large crystallites, manifested by the compressed shape of ISF in Fig. 3(a),(c). For small crystallites, β ~ 0.5 is observed as shown by the stretched shape of the ISF in Fig. 3(b),(d). A compressed exponential with β > 1 is indicative of a “jammed” system where local displacements create long-range spatial and temporal inhomogeneities^24–28^, while a stretched exponential with β < 1 is the hallmark of the dynamics of highly viscous liquids near *T*_*g*_, that corresponds to hierarchical dynamics with caging and escaping of groups of atoms/molecules via cooperative motion^29–31^.

## Discussion

For both samples, decay constant (τ) and exponent (β) are plotted in Fig. 4 and mean relaxation time are calculated from decay constant (see supplementary information section 7 for additional details). For small and large crystallites, decay constant of 1830 ± 50 s and 1130 ± 50 s was observed for Se sample at 32 °C, resulting in mean relaxation timescale of 5340 ± 50 s and 1020 ± 50 s. Recent rheological studies have indicated that the viscous flow is controlled by the Se-Se bond scission/renewal process with a characteristic timescale on the order of ~ 6310 s at 32 °C^32^. Calculated mean relaxation timescales for 32 °C as well as other temperatures (see Table S1 of supplementary information) are thus within the range of characteristic rheological timescales. For AsSe_4_ sample, a decay constant of 310 ± 50 s and 650 ± 150 s was observed at 84 °C resulting in mean relaxation timescale of 470 ± 50 s and 590 ± 150 s, for small and large crystallites respectively. The calculated mean relaxation timescale at 84 °C is consistent with the shear relaxation timescale of ~440 s, as obtained from the simple Maxwell model ${\tau }_{a}=\eta /{G}_{\infty }$, using ${G}_{\infty }$ of 5 GPa and *η* = 1000 GPa.s at 84 °C^23^. Here ${\tau }_{a}$ denotes the characteristic timescale for shear or α-relaxation, *η* is the viscosity for a given temperature and ${G}_{\infty }$ is the instantaneous (elastic) shear modulus^33^. Thus, the shear relaxation timescales are within the range of the calculated mean relaxation time from XPCS data for Se sample at all temperatures and AsSe_4_ sample at 84 °C, indicating that the dynamics at the crystal-liquid interface during the Se crystallization process is mediated via viscous flow of the supercooled liquid phase^7^. Here we note that for higher temperatures a significant deviation between the two timescales is observed for AsSe_4_ sample which will be addressed below.

In addition to crystal-liquid interfacial dynamics, another possible reason which could influence the observed dynamics is the motion of the crystallites in supercooled liquid surrounding the crystallites. However, this motion of crystallites is expected to increase at higher temperatures due to lowering of viscosity, which is inconsistent with the observation of slower decay constant (τ) at higher temperature for AsSe_4_ samples. Additionally, as discussed above, two different types of behaviour are observed based on size of crystallites for both Se and AsSe_4_ samples. The correlation function (*g*_2_) has a compressed shape for larger crystallites and stretched exponential shape for smaller crystallite as shown in Figs. 3 and 4. The two crystallite sizes are fairly close and thus, such a drastic change cannot be explained purely due to size-dependent motion of the crystallites in the super cooled liquid. Hence, for these Se nano-crystallites, the crystal-melt interfacial dynamics could play a critical role in crystallization and the fluctuations observed in the XPCS experiment could be due to atomic motions at the crystal-liquid interface, which are determined by the characteristic shear relaxation timescale of the liquid.

The slowdown of the fluctuation timescale and deviation from shear relaxation timescale at higher temperatures for AsSe_4_ can then be due to a change in the local chemical composition at the crystal-liquid interface. As the growth of the Se nano-crystallites proceeds, the Se content must get depleted at the crystal-liquid interface and the liquid in the surrounding region must get richer in As content. Higher As-content in the liquid results in higher viscosity and glass transition temperature^23^, consistent with the observed slowing down of fluctuation timescales. Based on the calculated mean relaxation timescale ~7000 s at 89 °C, we can estimate the viscosity for the higher As-content liquid using the Maxwell model to be *η* = 2.03 ×10^13^ Pa.s, indicating an increase in As content by ~5–7%. The fact that this slowdown is not observed in Se samples, emphasizes the role played by local chemical composition in determining the dynamical processes. On the other hand, for the large crystallites, the calculated mean relaxation timescales are: 590 ± 150 s at 84 °C and 1600 ± 300 s at 91 °C. Thus, the rate of increase in timescale with temperature is lower for the large crystallites and is consistent with the lack of the size dependence of these crystallites on temperature, as discussed above.

The drastic variation in the exponent (β) of the ISF for small and large crystallite is very surprising and point towards a difference in the mechanism of the crystallization process. As noted earlier, a stretched exponential decay of the ISF is typical of relaxation processes in glasses and supercooled liquids near *T*_*g*_^29–31^. The observation of a stretched exponential decay for small crystallites could indicate that the fluctuations observed for the Se nano-crystallites at their early growth stage occur at the crystallite/liquid interface which mediates the crystallization process. On the other hand, the compressed exponent observed for the large crystallites points towards a second mechanism. Compressed exponential behaviour has been observed in sputter deposition of amorphous thin films^34^ and in crystallization of metallic glasses^35^. This behaviour has been related to the nonlinearities in Kardar-Parisi-Zhang (KPZ) model of interfacial growth processes and scaling behaviour due to side-ways or lateral growth^36,37^. Screw dislocation assisted growth has been observed for Se crystallites, where the growth of the crystal results in the development of a spiral growth pattern or a screw dislocation and corresponds to lateral crystal growth^6^. The observation of a compressed exponential decay for the large crystallites may thus be indicative of such a situation where lateral growth becomes important and needs to be taken into account.

## Conclusion

We have presented XPCS measurements in chalcogenide glasses where x-ray beam induced crystallization was observed, providing us a unique opportunity to investigate the crystal-melt interface dynamics in supercooled Se and AsSe_4_ liquid near T_g_. An anomalous increase was observed in the fluctuation timescale at the crystal-liquid interface as the temperature is increased for AsSe_4_, which likely resulted from a change in the local viscosity at the interface, due to local compositional changes. In comparison, no slowdown was observed in Se samples and calculated relaxation timescales were within the range of rheological timescales, emphasizing the role played by local chemical composition in determining the dynamical processes during crystallization. Additionally, for large crystallites, a compressed exponential behaviour was observed, while for small crystallites, a stretched exponential behaviour was observed. This could be potentially related to the fact that for small crystallites the glassy dynamics occurring at the crystallite/liquid interface is dominant, while for large crystallites non-linear effects due to lateral growth need to be considered.

## Methods

### Samples

Se and AsSe_4_ glasses were synthesized from constituent elements (≥99.995% purity, metals basis) using the conventional melt-quench method. Elemental Se and As-Se mixture were melted at 400 °C and 650 °C, respectively, in evacuated (10^−6^ Torr) and flame sealed fused silica ampoules for 24 h in a rocking furnace. The melts were subsequently quenched by dipping the ampoule in water and the resulting glasses was annealed for 1 h at the nominal *T*_*g*_. Differential scanning calorimetry scan of this glass yields *T*_*g*_ corresponding to the onset temperature for glass transition to be ~35 °C for Se and ~88 °C for AsSe_4_. The glass samples were characterized prior to synchrotron studies by powder X-ray diffraction to ensure the absence of any crystallinity, and by Raman spectroscopy to ensure structural consistency with the literature reports.

### XPCS measurements

XPCS experiments were conducted at the Coherent Hard X-ray (CHX) beamline 11-ID at the National Synchrotron Light Source II (NSLS-II), Brookhaven National Laboratory. Disk-shaped samples (5 mm diameter, 1 mm thick) of Se and AsSe_4_ glass with polished flat surfaces were utilized for XPCS studies. Samples were heated on a hot plate inside a vacuum chamber (10^−5^ torr). The x-ray energy utilized was 12.8 keV, which is above the K-absorption edge of Se (12.658 keV). A coherent beam size of 3 µm was achieved by collimation with a set of 1D Be Compound Refractive Lenses and focused with a set of crossed Si kinoform lenses in front of the sample. The scattered beam was recorded with an area detector located 150 cm away from the sample with a 75 µm × 75 µm pixel size. When coherent x-rays scatter from the static or dynamic disorder present in the supercooled liquid sample, they acquire phase difference dependent upon the disorder and undergo constructive/destructive interference resulting in speckle patterns at the detector. This speckle pattern measured in k-space is a fingerprint of the sample structure in real-space. Moreover, if parts of the sample fluctuate in time, the observed speckle pattern will also fluctuate due to variations of the phase differences between scattered waves. These characteristic speckle patterns were recorded as a function of time, over a period of ~2 hours, at room temperature and at temperatures near *T*_*g*_. At each temperature, the sample was equilibrated for 30 minutes to ensure stabilization of the target temperature prior to collecting XPCS data for 2 hours. The temperature of the aluminium hot surface over which the sample is glued using a thermal conductive Silver paste is measured with a PT100 platinum resistance thermometer and controlled with a Lakeshore temperature controller. Additional information regarding thermal stability of XPCS setup is provided in supplementary section 1.

## Supplementary information

Supplementary Information.

## Data Availability

Data used and analysed in the study are available from the corresponding author upon reasonable request.
