# Supplementary material for: Dynamics at the crystal-melt interface in a supercooled chalcogenide liquid near the glass transition
Source: Sci Rep. 2020 Apr 3;10:5881. doi: 10.1038/s41598-020-62783-5 (PMC7125219; doi:10.1038/s41598-020-62783-5)
Supplement: Supplementary file 1 — Supplementary Information. [file 41598_2020_62783_MOESM1_ESM.docx]

**Supplementary Information:** **Dynamics at the crystal-melt interface in a supercooled chalcogenide liquid near the glass transition**

Jianheng Li^1^, Rahul Jangid^1^, Weidi Zhu^1^, Chris Kohne^1^, Andrei Fluerasu^2^, Yugang Zhang^2^, Sabyasachi Sen^1^, Roopali Kukreja^1^

1- Department of Materials Science and Engineering, University of California Davis, 1 Shields Avenue, Davis, CA 95616

2 – National Synchrotron Light Source II, Brookhaven National Laboratory, 743 Brookhaven Avenue, Upton, NY11973

1. **Stability of the experimental setup**


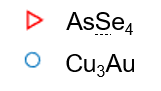

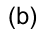
Thermal equilibration time of 30 minutes was utilized to minimize sample temperature gradients and the measurement was done in vacuum of 10^-5^ torr. The temperature variation as a function of time was tracked during the XPCS scan and was within 0.01 K. Additionally, as x-ray spot size (the sample volume probed) is 15 micron with a penetration depth of ~1 micron, we expect the temperature gradient to be negligible. Figure S1 shows XPCS stability scans performed to confirm the beamline stability at high and low temperatures. Figure S1(a) presents ISF of a reference Cu_3_Au sample at 140°C, and ISF of AsSe_4_ at 91°C. No significant variation in Cu_3_Au sample was observed showing the stability of beamline at high temperatures, and clearly showing that the decorrelation observed for AsSe_4_ corresponds to fluctuation in the sample itself. Figure S1(b) shows a room temperature XPCS scan for AsSe_4_sample. Again, no significant variation in correlation function ($g_{2}$) was observed.


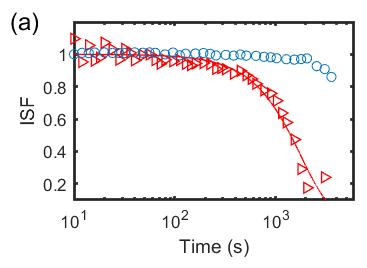

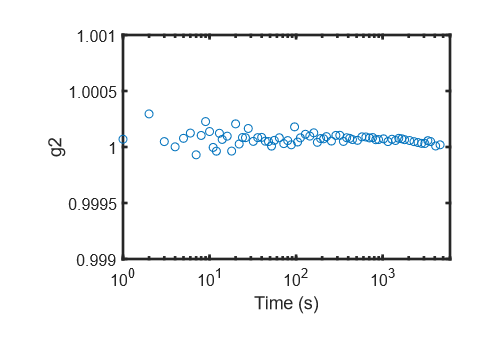


**Figure S1**: Stability of XPCS experimental setup, (a) ISF for reference Cu_3_Au sample at 140°C and AsSe_4_ at 91°C, (b) correlation function ($g_{2}$) for AsSe_4_ sample at room temperature.

1. **XPCS measurement at x-ray energy below Se K-edge**

In order to investigate the role played by the x-ray energy in beam-induced crystallization, we performed XPCS measurements at 7.35 keV at Beamline-8-ID-E of Advanced Photon Source (APS). This beamline provides access to x-ray energy lower than Se K-edge, allowing us to investigate the dynamics as a function of incoming x-ray energy. Figure S2 shows the polycrystalline ring due to beam induced crystallization, as well as evolution of correlation function ($g_{2}$) for large and small crystallites. While the total intensity measured at 8-ID was lower possibly due to different setup at two beamlines (NSLS-II and APS), we still observed beam-induced crystallization. Furthermore, the time constant (τ) and the exponent (β) measured for AsSe_4_ sample at 85 °C is similar to the dataset measured at NSLS-II. Specifically, β=1.5 is observed for large crystallites as shown in Figure S2 (b) and β=0.83 is observed for smaller crystallites as shown in in Figure S2(c), which is in agreement with NSLS-II datasets. The comparison between APS and NSLS-II data shows that while being above the Se K-edge results in higher absorption, the key results i.e. beam induced crystallization and associated dynamics are still observed for x-ray energies below Se edge.


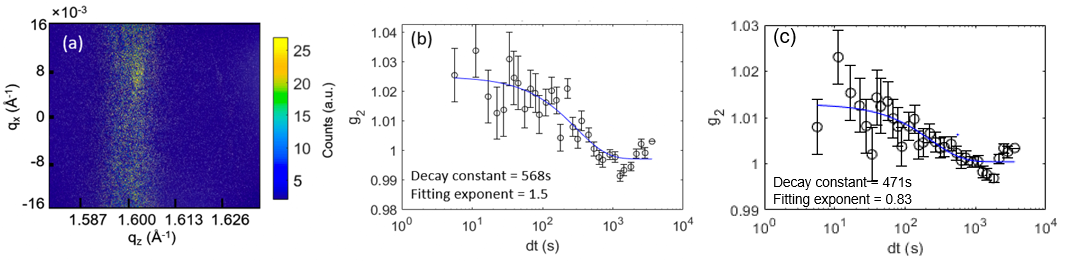


**Figure S2**: XPCS measurements performed at APS with x-ray energy of 7.35 keV, (a) polycrystalline ring observed at 85 °C, (b) correlation function ($g_{2}$) and fit with β=1.5 and τ = 568 s for larger crystallites, (c) correlation function ($g_{2}$) and fit with β = 0.83 and τ = 471 s for smaller crystallites. The values of β and τ are similar to that observed at NSLS with x-ray energy of 12.8 keV as discussed in the article.

1. **Crystallite size**

The peak width in x-ray diffraction measurements is given by convolution of instrumental and sample effects. In order to differentiate the two effects and accurately estimate the crystallite size, the instrumental broadening was measured using LaB_6_ powder which is used as a standard for peak width calibration in X-ray powder diffraction. The calibration was done using LaB_6_ powder with particle size of 10 μm, which shows negligible finite size effects. Following x-ray diffraction measurements of LaB_6_ powder, the full width at half maximum (FWHM) of 8.24e-4 Å^-1^ was obtained by Gaussian fitting after excluding the strain effect. The Bragg peaks for Se and AsSe_4_ samples were also fit using Gaussian function. For the convolution of Gaussian functions, the final width can be simplified to a simple square law,

(β_total_)^2^ =(FWHM_sample_)^2^ +(FWHM_inst_)^2^

This equation was used to estimate the FWHM due to sample effects and the crystallite sizes were estimated using Scherrer’s equation. For example, the smallest (largest) peak width, *β_total_*, measured in the experiment were 4.18e-3 Å^-1^ (1.810e-2 Å^-1^), which resulted in FWHM_sample_ of 4.11e-3 Å^-1^ (1.808e-2 Å^-1^) using FWHM_instr_ of 8.24e-4 Å^-1^. For large crystallites, a size in the range of 80-175 nm for Se sample and 70-105 nm for AsSe_4_ sample was obtained irrespective of the temperature of measurements. For small crystallites, a Se crystallite size of ~37 nm was obtained at 32 ºC, which increased to ~42 nm at 38 ºC. For AsSe_4_ sample, the crystallite size of ~35 nm was obtained at 84 ºC, which increased to ~50 nm at 89 ºC. The crystallite sizes reported were evaluated for the average data over an XPCS scan.

1. **Kymographs or ‘waterfall’ plots**

Figure S3 shows the intensity vs. time “waterfall” plots or kymographs for the measured intensity along a line cut across the polycrystalline ring for the (001) Bragg peak for both Se and AsSe_4_ samples. The Se data was taken at 38°C while AsSe_4_ data was taken at 91°C. From the kymographs, it is clear that the large crystallites move in and out of the Bragg diffraction condition during the 2 hour scan. The data analysis was done during the time period when the large crystallites are aligned to the x-ray beam as discussed in the next section. Here we note that, similar behavior was observed for all measured temperatures for small and large crystallites, and only representative kymograph plots are shown in Figure S3.


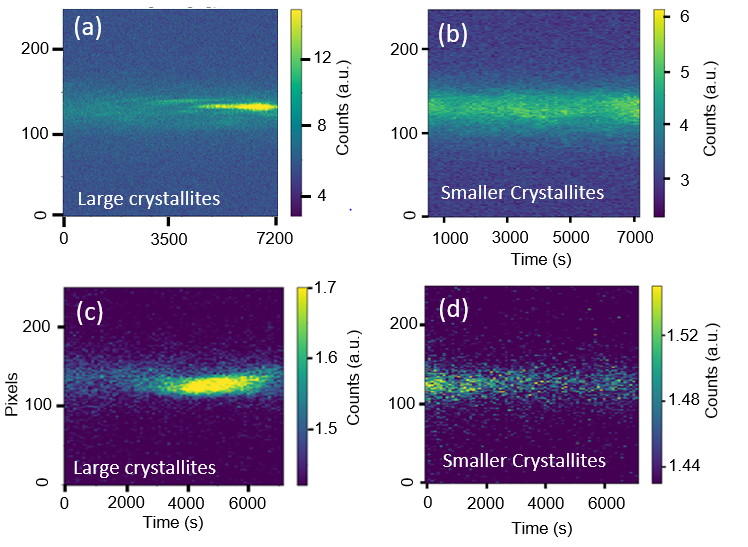


**Figure S3**: Kymograph or ‘waterfall’ plots showing the evolution of intensity as a function of time for a line cut along Qz of the polycrystalline ring, (a) and (b) Se at 38°C, (c) and (d) AsSe_4_ at 91°C for large and small crystallites respectively.

1. **ISF analysis for large crystallites**

The correlation function ($g_{2}$) for any large crystallite is calculated only during the period when that particular crystallite is aligned to the x-ray beam. For example, if the Bragg intensity due to larger crystallite appeared in frame 3000 and disappeared in frame 7000, as shown by the waterfall plot in Figure S4(a), only frames 3500 to 6500 were utilized for data analysis and calculation of the correlation function. This allowed us to eliminate inducing any artifacts in data analysis due to jump in intensities across the scan. Additionally, the first (last) 500 frames from 3000-7000 set were not utilized to minimize any influence due to appearance (disappearance) of the large crystallite on the measured decay constant. During this duration as shown in figure S4(b) the intensity of the Bragg peak is constant. Correlation function ($g_{2}$) and two time-correlation function are also shown in the figure S4. This indicates that at least during this period the crystal is oriented in the x-ray beam and the dynamics cannot be purely due to the motion of the crystallite in super-cooled liquid as discussed in the main article.


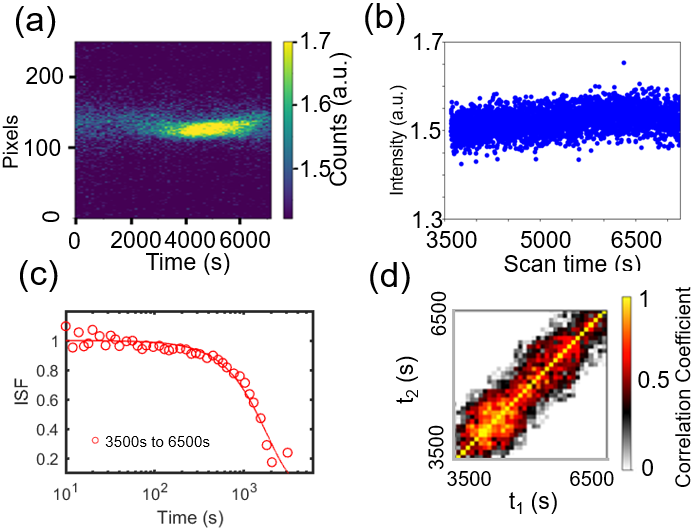


**Figure S4**: (a) Kymograph of ‘waterfall’ plots showing the evolution of intensity as a function of time for a large crystallite, (b) intensity as a function of time for the frames where large crystallite is aligned to Bragg condition, (c) correlation function ($g_{2}$), and (d) two-time correlation function calculated over the time window when large crystallite is aligned to Bragg condition as discussed in the text.

1. **Two-time correlation function and aging behavior**

Two-time correlation function, $g(q, t_{1}, t_{2})$, for Se and AsSe_4_ sample is shown in Figure S5 for temperature values of 36 °C and 91 °C respectively. Two-time correlation function allows us to directly capture the aging dynamics in an XPCS scan. It reflects the statistical similarity between average sample behavior measured at times $t_{1}$ and $t_{2}$, and is the time-resolved version of the correlation function ($g_{2}$). The center diagonal, where the two-time correlation function has highest intensity, corresponds to the elapsed time of the measurements. The intensity decreases as the difference between $t_{1}$ and $t_{2}$ grow i.e. off-diagonal. The width of the diagonal contour is directly proportional to $\tau$, the characteristic time scale (decay constant in the article) of the correlation function. No change in this diagonal width is observed over the measured time-period indicating lack of aging dynamics. Here we note that similar behavior for small and large crystallites were observed for all measured temperatures and only representative two-time correlation functions are shown in Figure S5.


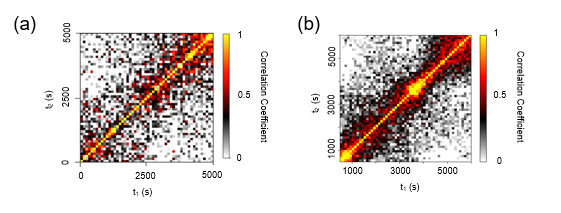


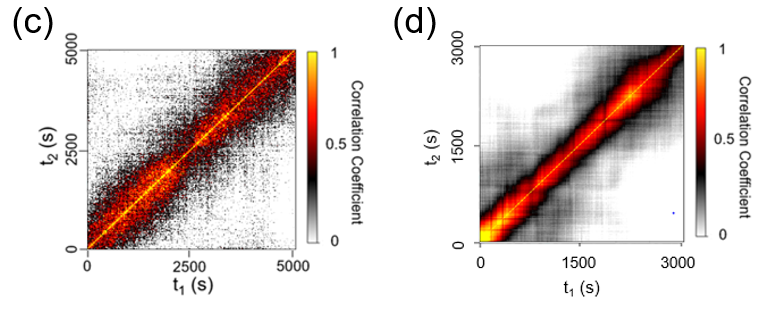


**Figure S5**: Two-time correlation function for (a) small, and (b) large crystallites for AsSe_4_ sample at 91°C and, (c) small and (d) large crystallites for Se sample at 36 °C

The normalized *g_2_(q,t)* function, extracted from two-time correlation function for two different waiting times, *t_w_*, for both small (a-d) and large (e-h) crystallite is shown in Figure S6 and S7 for Se and AsSe_4_ sample, respectively. For the large crystallites, the starting time and duration depend on specific time when the large crystallite are aligned to the Bragg condition. As mentioned above, any differences in the correlation function between the two waiting times would represent aging behavior, i.e. the change in material response as a function of time after equilibration at a certain temperature. The fact that the correlation function with two different waiting times are similar and show no trend as a function of temperature for both Se and AsSe_4_ samples indicate that no aging dynamics is observed.


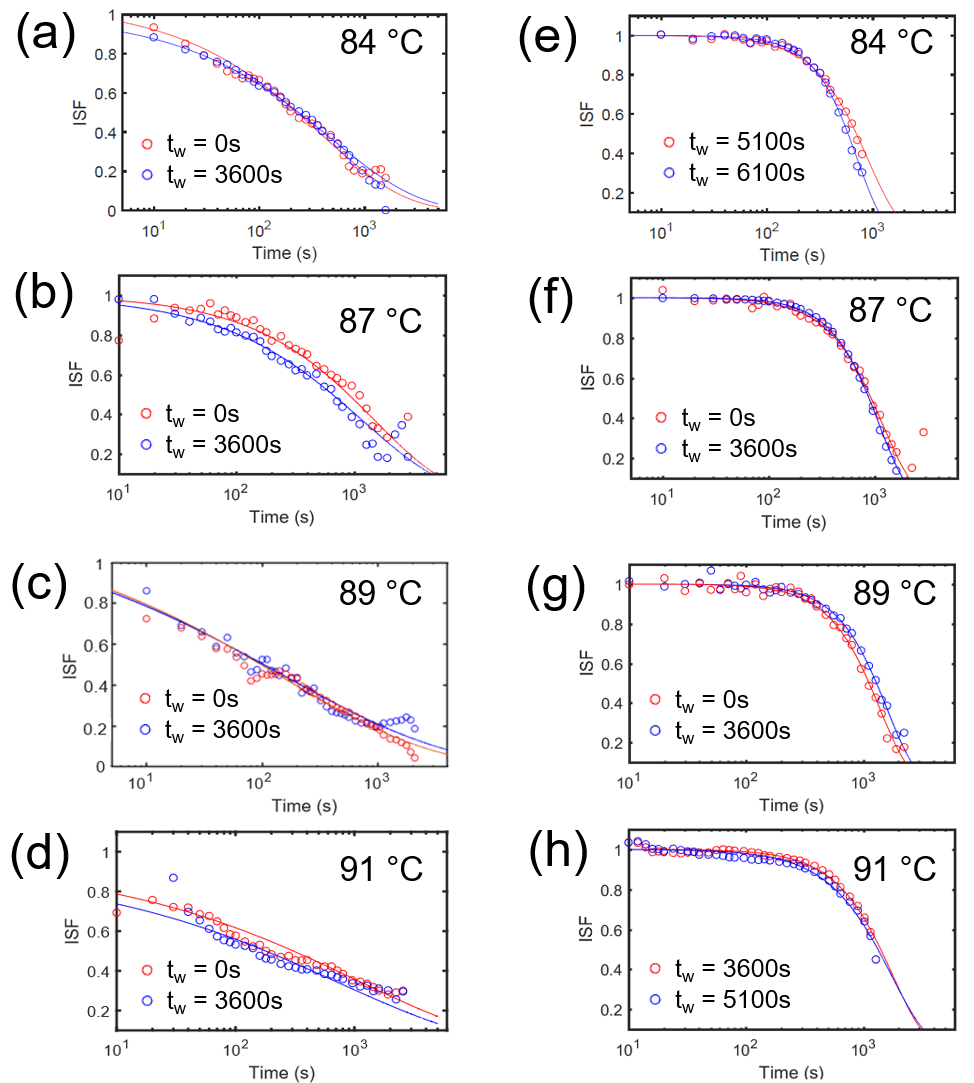


**Figure S6:** Aging behavior for (a)-(d) small crystallites, and (e)-(h) large crystallites for Se sample for various temperatures as a function of waiting time, *t_w_*.


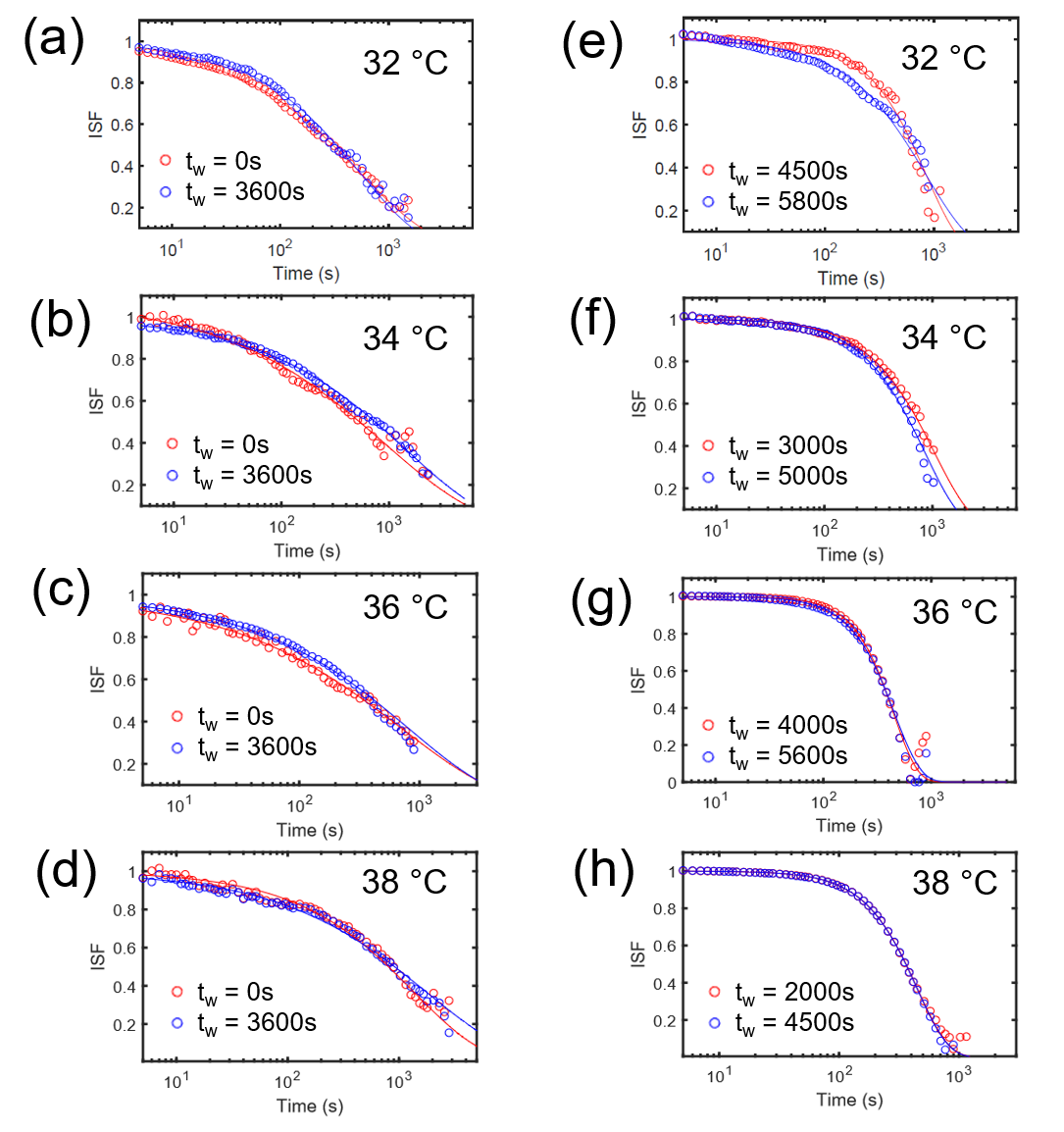


**Figure S7**: Aging behavior for (a)-(d) small crystallites, and (e)-(h) large crystallites for AsSe_4_ sample for various temperatures as a function of waiting time, *t_w_*.

1. **Mean relaxation timescales and comparison with shear relaxation timescales**

The mean relaxation time ($<\tau>$) was calculated using decay constant and the stretching exponent using the following equation [Reference: R. K. June *et al.* Biomed Eng Res. 2(4), 153–158 (2013)],


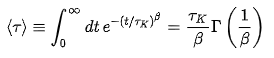


where, β is the exponent, $\tau_{K}$ is the decay time and Γ represents gamma function. The decay constant, stretching exponent as well as calculated mean relaxation timescale for both types of crystallites is included in table S1 for Se and AsSe_4_. The rheological relaxation timescales from the literature [Ref 23, 32 and 33 of the article] are also included in the table for the comparison.

|  | Small Crystallite | | Large Crystallite | |  |
| --- | --- | --- | --- | --- | --- |
| T (°C) | τ (s) | Mean relaxation time (s) | τ (s) | Mean relaxation time (s) | Rheological timescale (s) |
| Se |  | | | | |
| 32 | 1830±50 | 5340±50 | 1130±50 | 1020±50 | 6310 |
| 34 | 2430±50 | 3030±50 | 1195±50 | 1070±50 | 4450 |
| 36 | 2075±50 | 3490±50 | 465±50 | 420±50 | 3550 |
| 38 | 1950±50 | 3030±50 | 550±50 | 495±50 | 2510 |
| AsSe_4_ |  | | | | |
| 84 | 310±50 | 470±50 | 650±150 | 590±150 | 440 |
| 87 | 1620±100 | 3250±100 | 945±150 | 845±150 | 195 |
| 89 | 2775±100 | 7050±100 | 1400±150 | 1300±150 | 115 |
| 91 | 3450±300 | 12440±300 | 1800±150 | 1620±150 | 70 |

Table S1: Decay constant ($\tau)$ and stretching exponent ($\beta$) obtained from ISF fits were used to calculate mean relaxation timescale for both types of crystallites. Rheological relaxation timescales as a function of temperature are also included.
